# Data-Driven Compensation for Flow Cytometry of Solid Tissues

**DOI:** 10.1155/2011/184731

**Published:** 2011-09-06

**Authors:** Nickolaas Maria van Rodijnen, Math Pieters, Sjack Hoop, Marius Nap

**Affiliations:** ^1^Department of Pathology, Atrium Medical Centre, P.O. Box 4446, 6401 CX Heerlen, The Netherlands; ^2^TiCC, Tilburg University, Warandelaan 2, 5037 AB Tilburg, The Netherlands

## Abstract

Propidium Iodide is a fluorochrome that is used to measure the DNA content of individual cells, taken from solid tissues, with a flow cytometer. Compensation for spectral cross-over of this fluorochrome still leads to compensation results that are depending on operator experience. We present a data-driven compensation (DDC) algorithm that is designed to automatically compensate combined DNA phenotype flow cytometry acquisitions. The generated compensation values of the DDC algorithm are validated by comparison with manually determined compensation values. The results show that (1) compensation of two-color flow cytometry leads to comparable results using either manual compensation or the DDC method; (2) DDC can calculate sample-specific compensation trace lines; (3) the effects of two different approaches to calculate compensation values can be visualized within one sample. We conclude that the DDC algorithm contributes to the standardization of compensation for spectral cross-over in flow cytometry of solid tissues.

## 1. Introduction

Multiparameter flow cytometry (MP-FCM) of solid tumors is a powerful tool for quantification of antigen expression and DNA content, based on large numbers of individual mammalian cells. However, simultaneous application of different fluorochromes introduces spectral cross-over. Spectral cross-over is the acquisition of fluorochrome intensities from a primary fluorochrome in the detector(s) used to acquire the intensity of secondary fluorochromes. Compensation is the estimation of the amount of fluorochrome intensity that needs to be subtracted from the acquired intensities to correct for spectral cross-over [1–4]. Proper compensation is achieved when the compensated data in the cross-over detector has no bias in the fluorescence distribution that is related to the intensity measured in any other detector [5]. To achieve proper compensation, the amount of spectral cross-over of each fluorochrome in a flow cytometry panel can be estimated with a single stained control (SSC). An SSC consists of a single cell suspension of which the individual cells are labeled with only one fluorochrome. This fluorochrome, of which the intensity is acquired in its primary detector, exhibits spectral cross-over in other secondary detectors. The cross-over of the SSC in each secondary detector is expressed as a percentage of the intensity acquired in the primary detector. This percentage is based on the correlation coefficient between the fluorochrome intensity of a SSC in the primary and secondary detector(s) [1, 3]. The combination of all the percentages cross-over for each SSC, in each secondary detector, is expressed in a compensation matrix. It is common to calculate this compensation matrix once or twice a day and to use it for all following acquisitions.

The problem of flow cytometry when working with cells originating from solid tissues is the use of propidium iodide (PI). PI is a dye that binds to DNA, and the acquired intensity is proportional to the amount of DNA in a (tumor) cell. The major advantages of PI are that (1) the DNA profile of the acquired (tumor) cells can be studied in relation to their phenotype, (2) it is possible to get very small coefficients of variation (CV) even in paraffin embedded material, in contrast to other DNA dyes like TOPRO3 and, (3) PI doesnot stick to the interior of the flow cytometer, like DAPI does. The major disadvantages of the PI dye are (1) spectral cross-over in all detectors that primarily detect fluorochromes excited with 488 and 635 nm lasers and (2) it binds noncovalently to DNA. The loose binding makes compensation case specific because the average amount of bound PI is also dependant on the number of cells in a single-cell suspension. When the number of cells varies from case to case, the acquired average intensity of PI varies, and therefore the primary detector of PI needs variable amplification. Variable amplification of detectors in a flow cytometry system disturbs compensation matrices. Therefore each case needs its own compensation matrix. As a consequence compensation needs to be performed for each case separately. This is time consuming and therefore costly.

In this paper we investigate spectral compensation using an algorithm we called Data-Driven Compensation (DDC) which is especially developed to deal with variable compensation matrices when PI is used to study the DNA content of (tumor) cells in a single-cell suspension. We describe the analysis steps of the automated compensation concept based on the data of a 2-color experiment. In 2-color flow cytometry each event or count represents the acquired fluorochrome intensities for the 2 colors of one cell. The main difference between the DDC method and known compensation algorithms is based on the fact that DDC calculates the compensation values on automatically selected counts. The key characteristic of the selected counts is that they all have the same primary fluorescence. This feature reduces the value spread of the counts that are selected to calculate the correlation between the acquired fluorochrome intensities in the primary PI detector and a secondary detector. The reduced spread of these counts thus opens the possibility of automatically compensation for spectral cross-over from PI, avoiding the need for any manual intervention.

## 2. Material and Method

### 2.1. Patient Population

Over the period of January through December 2007 we analyzed 227 lymph node biopsies that were obtained from sentinel lymph node (SLN) procedures in breast cancer patients [6]. The SLNs were routinely examined at 3 levels with steps of 500 *μ*m by a pathologist for (micro)metastasis. Each examined level consisted of a 3 *μ*m, H&E stained section and processed for histology. These 3 levels are reached by taking 10 sections of 50 *μ*m before the next H&E level. All four 50 *μ*m sections were collected in a glass tube and processed for flow cytometry. For a detailed description of this procedure see [6, 7]. In brief, a single cell suspension of individual lymph node cells was generated from the four 50 *μ*m sections and divided in two separate samples; one served as negative control (NC) and was incubated with 2 *μ*L of a nonrelevant mouse monoclonal antibody isotype IgG1 (X 0931, DAKO; diluted 1/20); the second one served as test sample (TEST) and was incubated with 2 *μ*L of a cytokeratin monoclonal antibody isotype IgG1 (clone MNF116; M 0821, DAKO; diluted 1/20, DAKO). The nonrelevant mouse monoclonal antibody served as isotype negative control to determine the amount of nonspecific antibody binding, and the cytokeratin antibody labels epithelial metastatic cells in the TEST. After overnight incubation the two single-cell suspensions were washed twice with PBS (pH 7.4) and incubated with a polyclonal goat antimouse FITC (F 0479, DAKO, undiluted) for 1.5 hours, followed by another two washing steps. After these last washing steps 1 mL PI (1 *μ*g/mL, Sigma) was added for DNA labeling. When the antigen expression detected via the FITC signal in the TEST sample passed a given threshold (1% positive events), a sample was considered positive for metastasis, based on previous experience [8]. The height of the threshold was set on the NC and contained exactly 0.5% FITC-positive events above this line. In the positive cases the acquired DNA histogram provided information about the ploidy status of the metastasic material in relation to the FITC-positive events.

### 2.2. Data Set

To ensure comparability of individual cases data files, 190 of a total of 227 data sets were selected based on a minimum of 100.000 counts acquired for both the NC and TEST files. A further 38 cases were excluded from the data set because of a laser replacement and laser beam alignment optimization, leaving a total of 152 cases to enter the final data set.

### 2.3. Data Acquisition

All flow cytometric acquisitions were performed on a BD FACSCalibur (BD Biosciences, San Jose, CA) flow cytometer with a single 488 nm argon laser. Forward light scatter, right-angle (side) scatter, and two fluorescence signals (FITC and PI) were acquired simultaneously in list mode. The fluorescence was measured using the standard photomultipliers (PMTs) and optical filters (530/30 nm BP filter for FITC and 670 nm LP filter for PI). The forward scatter was recorded with a photo diode. For FITC emission the pulse height was recorded (FL1h), for PI emission, in addition to the pulse height (FL3h), also the pulse width (FL3w), to calculate the area (FL3a), was acquired.

For each sample 100.000 counts were acquired, triggered on FL3. The DNA content was recorded in linear mode with a resolution of 1024 units. The FITC expression of the cells was recorded in logarithmic mode with 4 log decades in a range of 10^0^ to 10^4^, also using a resolution of 1024 units. No hardware compensation was performed during flow cytometric acquisition.

### 2.4. Data Analysis According to Standard Available Procedures

For the analysis of the flow cytometric data files the software package Summit V 4.0. (DakoCytomation, Glostrup, Denmark) was used. Selection of single cells was accomplished by setting a region in a dot plot of FL3-w (abscissa) against FL3-a (ordinate) of the PI parameter for the NC and TEST separately. As a standard procedure, the height of the single cell region was set to include the 2C, 4C, 6C and 8C ploidy clusters. These four clusters represent cells in, (1) the diploid G0/G1 (2C) mode; (2) the combined G2M-phase (4C)—diploid cell cycle—plus tetraploid G0/G1 cells; (3)-(4) two aggregate peaks (6C and 8C), with the 8C peak containing cells in the G2M phase of the tetraploid cell cycle. The four clusters are identified in Figure 3(a). If an aneuploid population with a DNA index (DI) above value “2” would be present in the FL3-a DNA histogram, the height of the single-cell region could be expanded to include the aneuploid G2M peak. This aberration could not be identified in the present dataset. There is no objective criterion to set the width of the single-cell region [9], and therefore it is determined by the operator. However, analysis revealed that all applied single-cell regions include 70–80% of the total amount of counts, both for the NC and the TEST. 

In the standard procedure, not data-driven, compensation for spectral cross-over is accomplished using a dot plot of FL3a-PI (abscissa) against FL1h-FITC (ordinate), by entering a percentage (compensation value) in the compensation matrix of the Summit software. As stated before, correct compensation is achieved when the compensated FITC intensity has no bias in the fluorescence distribution that is related to the acquired PI intensity. Since spectral compensation leads to compensation artifacts [1, 3, 5, 8, 10], commonly the median values of individual cell clusters are compared to determine the amount of intensity related bias in compensated dot plots. However, in PI acquisitions around 80% of all the counts are located in the 2C cluster. Therefore we choose to compare the median value of the 2C cluster with the median value of the combined counts in the 4C, 6C, and 8C clusters. Therefore the compensated dot plot is divided in 2 regions that are separated by a vertical line. This line is set to separate the 2C cluster on the left hand from the 4C, 6C, and 8C clusters on the right hand. This region setting serves in comparing the median values obtained for each region of the dot plot. When the two median values differ too much after operator activation of the compensation value, this initial value is adjusted and the compensation operation is performed again. As indicated already above, after compensation of the NC sample, a horizontal cut-off line is set in the dot plot, allowing no more than 0.5% FITC-positive counts to exceed this threshold value. The same compensation settings are applied to the TEST sample data. If the compensated TEST data show an increased or decreased median value for the 2C cluster as compared to the median value of the combined 4C, 6C, and 8C clusters, the compensation value is adjusted, before applying the same cut-off line as defined above. In our hands, this adjustment was only required in cases with large metastasis (>2.0 cm) resulting in a higher intensity of the FITC signal. When the percentage of positive cells (counts above the cut off line) in the TEST sample exceed 1.00% the sample is considered positive. The following data were collected: (1) the compensation values for the NC and TEST samples and (2) the percentage of MNF116-positive cells (counts above the threshold) in the TEST sample, (3) DNA ploidy.

### 2.5. From Standard Compensation Procedures to Data-Driven Compensation

This section describes the theoretical background that stimulated us to develop a data-driven approach for compensation of spectral cross-over compared to hardware-driven or operator-dependant methods. The total fluorescence intensity detected by a primary fluorescence PMT in a 2-color flow cytometer setup consists of four contributions: (1) the fluorescence signal of the primary fluorochrome, (2) the cellular autofluorescence [11], (3) the optical and the electronic noise generated in the cytometer [4, 5], and (4) the cross-over signal from the secondary fluorochrome [1] also called the *intensity-dependent bias* [5]. The aim of the compensation step is the determination of the sole contribution of the primary fluorescence, within the total fluorescence intensity measured. Autofluorescence and noise contribute to the variance of the overall fluorescence signal. The autofluorescence also increases the intensity of the overall fluorescence signal. Both autofluorescence and noise are inevitable. They can be minimized, as we will show later, but cannot be offset by a mathematical compensation. In contrast to this complexity, the estimate of contribution of the cross-over to the total signal intensity can readily be performed by an experienced operator by visual interaction. 

To confirm and visualize this intensity-dependent bias, operators plot the fluorescence intensities as detected by the primary PMT against those detected by the secondary PMT. The resulting dot plot contains an approximately linear distribution of points with the intensity-dependent bias expressed as the slope of the distribution [1]. It is thus key to define or calculate the value of this slope of the intensity dependent bias in order to apply proper compensation.

Most dedicated software packages such as Summit version 4.0 (Dako Cytomation), Flowmax V 2.2 (Partec), and WinList V 5.0 (Verity Software House Inc.) function with manually determined values of the intensity dependent bias. In contrast, the semiautomatic determination of the compensation value is used as a feature in the Winlist, Summit, and FloJo (V 7.5) software packages. All of these methods essentially work with an initial estimate of the intensity dependant bias and require operator evaluation and validation through visual inspection of the compensation results. In most cases this process results in an iterative manual refinement of the initial estimate of the intensity dependant bias and is very clearly operator dependent.

In this paper we propose a fully automated compensation algorithm we have called Data-Driven Compensation or DDC. With this method we aim to exclude operator-associated subjectivity in defining the compensation value. To achieve this, we select for a subset of counts with the common characteristic of value “zero” for their primary fluorescence. As defined above, the background fluorescence is composed out of three factors only: autofluorescence, optical or electronic noise, and cross-over from a second fluorochrome. Autofluorescence and noise are independent of the signal of a second fluorochrome. The cross-over contribution however increases the intensity of the observed fluorescence proportionally to the intensity of the second fluorochrome. This difference in contribution between “signal-independent” and “signal-dependent” elements provides for a good tool to measure the amount of cross-over. If cross-over signaling would not exist, all counts with zero primary fluorescence would effectively be displayed on a line parallel to the abscissa or ordinate in a dot plot. Therefore, the slope of the total fluorescence in the selected counts with zero primary fluorescence can serve this purpose. The resulting selected subset has a reduced variance of FITC intensity when compared to that of all of the counts acquired as illustrated in Figures 3(a) and 3(b). 

In the following section we will describe formal approach of compensation, using the DDC concept, and explain the automatic determination of the intensity dependent bias.

### 2.6. Formalization of Compensation

In a 2-color flow cytometry experiment with single cross-over, we define a detector *D_A_,* sensitive to a range of wavelengths of a specific fluorochrome *A*, and a detector *D_B_*, sensitive for fluorochrome *B*. Due to the spread of wavelengths emitted by fluorochrome *B*, there is cross-over (or spillover) of value *S_BA_* of fluorochrome *B* into the detector of fluorochrome *A*. The goal of compensation is to correct for this cross-over. The equations for the signals picked up by the two detectors are generally expressed as [1, 12]

(1a) $$\begin{array}{c} D_{A}=A+S_{BA}B, \end{array}$$

(1b) $$\begin{array}{c} D_{B}=B+S_{AB}A. \end{array}$$

These equations can be rewritten to compute the actual (or compensated) fluorochrome value for *A*:

(2a) $$\begin{array}{c} A=\frac{D_{A}-S_{BA}D_{B}}{1-S_{BA}S_{AB}}, \end{array}$$

(2b) $$\begin{array}{c} B=\frac{D_{B}-S_{AB}D_{A}}{1-S_{BA}S_{AB}}. \end{array}$$

Since we have defined *S_AB_*  =  0, there is no cross-over from *A* into *D_B_*. Therefore these two equations can be rewritten as

(3a) $$\begin{array}{c} A=D_{A}-S_{BA}D_{B}, \end{array}$$

(3b) $$\begin{array}{c} B=D_{B}. \end{array}$$

### 2.7. DDC

In essence the DDC algorithm automatically selects counts with zero primary fluorescence content from the acquired flow cytometry data. Below we describe the automatic selection of the zero fluorescence counts and the calculation of the cross-over values from these counts.

**Figure 1:**
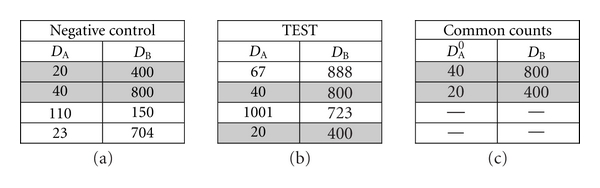
Identification of the common counts as basis for the calculation of the cross-over value. The matrices for the NC (a) and TEST (b) result from an experiment with 4 counts (*N*) and a single cross-over from fluorochrome B in detector *D_A_*. The matching rows of the NC and TEST matrices (grey) are defined as the matrix of the Common Counts (c). The rows in the Common Counts define counts with zero primary fluorescence for fluorochrome *A* (*D*_*A*_^0^). The cross-over value from fluorochrome B in *D_A_* can be calculated directly from the common counts. In the case as shown, 40/800 = 0.05 and 20/400 = 0.05.

As described, an NC and a TEST sample, with *N* = 100.000 counts each, were acquired in every SLN experiment. As shown in Figure 1, each acquisition can be represented by a matrix of 2 merged vectors, *D_A_* and *D_B_* of length *N*. The common counts (CCs) are defined as the matching rows (vectors of length 2) of the matrix of the NC sample and of the TEST sample. As shown, the common counts are those counts for which the total fluorescence of both fluorochromes *A* and *B* is identical in the NC and the TEST matrices.

In the files every count consists of a total fluorescence signal to which different components contribute. In a similar way the total number of counts is made up of different classes of counts. Three types can be identified in the NC file: (1) negative counts with zero primary fluorescence, (2) positive counts with primary fluorescence signal generated by nonspecific binding of nonrelevant mouse Ig (staining background), and (3) counts that solely result from noise and other flow cytometer imperfections. In a typical experiment less than 5% of type 2 counts are expected from the immunostaining in the NC. In a well-calibrated and maintained flow cytometer, the number of type 3 counts will be less than 0.1%. In summary, the counts of the NC sample consist of about 95% of the type (1) counts.

The counts collected from a TEST sample consist of the same three types as those described for the NC sample, supplemented with counts that contain sufficient fluorescence intensity to be defined as “positive.” The common counts of the NC and TEST matrices will *not* include these “positive” counts, as they appear exclusively in the TEST sample files. In the common counts, any positive counts therefore must be of type (2). The expected amount of type (2) counts maximize about 5% of the total counts. In conclusion, the majority of the common counts contain values of zero primary fluorescence for both the NC and TEST files.

Having identified the common counts with zero primary fluorescence, we can now proceed to calculate the cross-over values. In the case of single cross-over from *B* in *D_A_*, only an NC with a nonrelevant Ig coupled to fluorochrome *A* is needed. Each count in the CC consists then of two values: (1) an intensity value for *A* (*D_A_*) which holds the values for zero primary fluorescence (*D*_*A*_^0^) and (2) an intensity value for *B* (*D_B_*). The cross-over value *S_BA_* can then be calculated from the CC with the rewritten (3a):

(4) $$A=D_{A}^{0}-S_{BA}D_{B}=0\Rightarrow S_{BA}=\frac{D_{A}^{0}}{D_{B}}.$$

These equations show that the cross-over value S_BA_ can be calculated from the CC. For the experiments completed in this work only (4) was used. Based on the selection of the dyes, we know that only cross-over from fluorochrome *A* (PI) into* D_B_* can be shown but no spillover of fluorochrome *B* (FITC) into *D_A_* is present. Under these conditions, data analysis can be limited to the NC using a nonrelevant, FITC-labeled antibody. As such the CC matrix will consist of counts with only zero primary fluorescence for *D_A_* (FITC detector). For two-color experiments with double cross-over, the formula to calculate the cross-over values (*S*_*BA*_ and *S*_*AB*_) can be found in Appendix A.

### 2.8. Correctness of Compensation

Even though we were able to present a proper path to define compensation, no objective measure exists to quantify and validate the correctness of compensation. Visual inspection is subjective as it depends solely on operator experience while the RIDB value is also visually estimated from the compensated dot plots. Herzenberg et al. [4] proposed displaying the data on a logarithmic scale [13] and compared the centre position of the stained population with the centre position of the unstained cell population (background). Expected positions of the centres of the unstained (sub)populations in a correlated fluorescence plot can be described along the respective axes. These positions might be indicative for over- or under-compensation. However, a positive TEST (sub)population will always have a centre “above” the unstained (background) population along the same intensity axis, regardless of a proper compensation setting.

## 3. Results

To test the DDC algorithm we conducted three experiments. In the first experiment we compared the compensation values obtained with DDC to those of manual compensation. All manual compensations were performed individually in the Summit software. We consider the performance of the DDC algorithm comparable with manual compensation when the two sets of compensation values agree. In the second experiment the results of the SLN analysis are calculated. These results are expressed as the percentage of positive counts in the TEST sample. The outcome is compared between the DDC and Summit paths. For these first two experiments a new MATLAB implementation was written for the reanalysis of the cases in the DDC dataset, which used the function FCA_readfcs [14]. The only difference between the automated Matlab implementation and the manual analysis in Summit software is the compensation algorithm. The Matlab implementation of DDC includes the “Trust Region” algorithm [15–17]. This algorithm calculates the optimal fit of the compensation trace line through the CC. Alternatively, the manually determined compensation trace line, using the Summit software, is based on a visually determined optimal fit through all available counts. After compensation is completed, a doublet discrimination step is applied. This doublet discrimination will not influence the first experiment, although it slightly affects the percentage positive counts in the second experiment.

The third experiment is performed to express the correctness of compensation. We herewith propose a new option, building on earlier work. Two methods have been suggested to achieve proper compensation. The first method uses a single stained control (SSC) for each fluorochrome in the panel [1, 3, 4, 12, 18]. The second method uses an isotype control only [2]. In our 2-color experiments we have only a cross-over from PI in the FITC channel. This setup compares more with the second method (isotype control—implemented as NC) than with the first (SSC method—sample containing only PI). As described above, within the NC, we expect less than 5% positive FITC counts in this sample (background signal), the observed slope (or trend line) therefore of the CC from a NC and SSC should be approximately the same as the slope (or trend line) observed of the CC from a NC and TEST. When the two slopes agree, the calculated compensation using the DDC procedure leads to a correct compensation value. Since we do not routinely apply an SSC for the sentinel test in our laboratory, we acquired an additional 45 cases with an extran SSC (containing only PI) for the purpose of this third experiment. 

### 3.1. Comparative Evaluation of the Compensation Values

**Figure 2:**
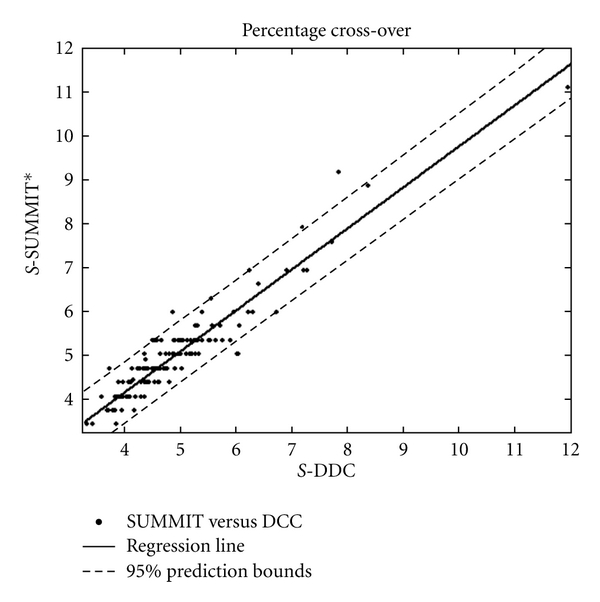
Results of a regression analysis of the manually determined compensation values using Summit V4.0 (*S*-SUMMIT, ordinate) and the automatic compensation values calculated using the DDC algorithm (*S*-DDC, abscissa), for the negative control samples. All compensation values are calculated on 100.000 cellular events. A regression line (black solid line) is fitted through all the admissible data points. The dashed lines represent the 95% prediction bounds of the fit.

To test the capability of the DDC algorithm to correctly calculate the compensation values, we compared these calculated values against the manually determined values. After linear transformation of the Summit compensation values *S*_SUMMIT_ into appropriately rescaled values *S*_SUMMIT*_ (see Appendix B), the linear relation between the paired compensation values (Summit and DDC) is defined by the regression line

(5) $$S_{{\text{SUMMIT}}^{*}}=0.90,\;\;S_{\text{DDC}}+0.0049$$

as illustrated in Figure 2. This figure shows the rescaled manually determined compensation values *S*_SUMMIT*_ (on the ordinate) against the compensation values (*S*_DDC_) generated by DDC (on the abscissa). The dots indicate the paired compensation values, expressed as the percentage cross-over for each case in the dataset. The combination of the high correlation (*R*-square = 0.90) with the limited scatter around the regression line (Root Mean Squared Error = 0.0034) indicates that the DDC method generates compensation values that are very comparable with those determined manually. As indicated by the regression slope of 0.90, the DDC-generated compensation values are slightly higher than the values generated by the Summit software. The horizontal clustering of the “Summit value” dots in the graph reflects the need of an operator to resort to rounded compensation values upon manual interaction.

A total of five cases (out of 152) fall outside the 95% confidence interval. Two of these five cases are very close together under the lowest prediction bound, and their mutual characteristic is high background signal of the NC sample. This high nonspecific binding in these NC samples leads to an increase of CC values. Since this nonspecific reactivity induces a shift towards higher FITC intensities, fitting the compensation trace line leads to a higher compensation value, and thus to overcompensation. The other three cases (above the highest prediction bound) were reanalyzed by the two operators. They both turned out to be manually overcompensated in the Summit procedure.

### 3.2. Comparative Evaluation of the TEST Results

The objective of the SLN procedure is to determine the percentage positive counts in a TEST sample compared to its NC. Therefore a second regression analysis was performed to compare the percentage positive counts found with the original SLN Summit procedure (pos_Summit) versus the procedure with incorporation of the DDC algorithm (pos_DDC). The regression line is defined by

(6) pos_Summit=0.90∗pos_DDC+0.042.

Also in this set, a good match between the paired results was found. The high correlation value (*R*-square = 0.98) and the limited scatter around the regression line (Root Mean Squared Error = 0.084) indicate that the results of the analysis with using the automated DDC method are very comparable with the results from the manual compensation method. Only six cases out of the 152 fall outside the 95% confidence interval. Reanalysis of these six cases by the two operators revealed four cases for which lower Summit-based compensation values were used for the NC as compared to the TEST. Over-compensation of the TEST data resulted in a lower percentage of positive counts. This compensation error is a common artifact and well documented in the literature [1, 5]. The remaining two cases were correctly compensated by the DDC algorithm as re-analysis with the Summit software revealed that both cases were indeed under-compensated through visual inspection. It appeared that with applying just 0.1% additional compensation decreased the height of the threshold in the NC sample. This operation resulted in an increase in the percentage of the positive counts in the TEST sample, minimizing the differences between the Summit and the DDC algorithm outcomes.

### 3.3. The Correctness of Compensation

Even though compensation values might agree between the two different methods applied, it doesnot automatically imply that these compensation values are correct. As mentioned before we acquired an additional 45 cases, stained according the SSC concept. The compensation values found with using the CC of the SSC and the NC (set 1) can thus be compared with the ones obtained using the CC of the NC and TEST (set 2).

The 2 sets of compensation values show no Gaussian distribution and are highly correlated (*R*-square = 0.9651, RMSE = 1.5 × 10^−3^). The spread between the maximum and minimum compensation values in both sets is comparable, 4.3% in the first set and 4.2% in the second set. There is no statistical evidence for different compensation values in the two sets, based on 45 cases. However, all the compensation values, except one, in the first set were lower than their matching compensation values in the second set. This difference is also reflected in the mean compensation values, which are 4.9% for the first set and 4.7% for the second set. Despite the statistical insignificant compensation values between the two sets, the visual effect of applying two different compensation values to the same sample is evident.

**Figure 3:**
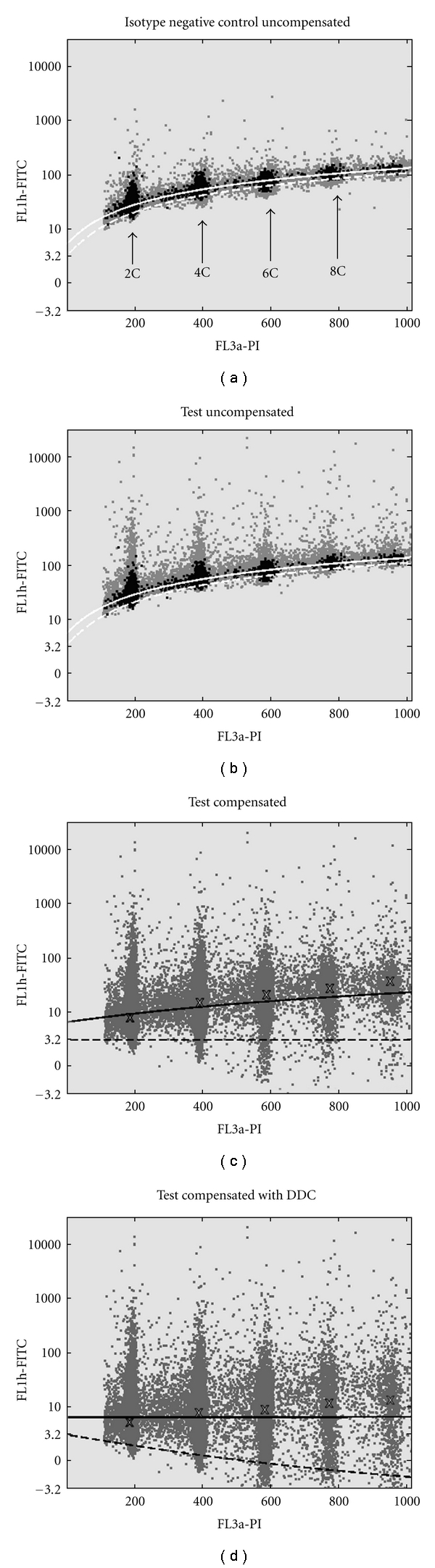
(a) shows events of a negative control (NC). (b) shows the events of the matching TEST. Both graphs are uncompensated 2-color plots, from sentinel lymph node (SLN) tissue. (c) shows the TEST after compensation, using a trace line fitted through the common counts of the NC and SSC (dashed line). (d) shows the TEST values, after compensation with a compensation trace fitted through the CC of the NC and the TEST (the full line). Each dot plot consists of 100.000 counts. The grey dots represent the individual counts in each dot plot. The black dots represent the common counts. The median values of each of the 5 distinct clusters (ploidy level 2C, 4C, 6C, 8C, and 10C) are marked with an “X.” For optimal visual representation, the pairs of compensation trace lines are white in the upper graphs and black in the lower graphs. Note that the 2 different compensation trace lines seem to be parallel in (a) and (b), which is an optic illusion because the ordinate is logarithmic. In the linear domain these 2 compensation trace lines diverge. The dot plots represent logical (*T* = 10000, *W* = 0.5, *M* = 4.5) [13], FITC fluorescence (ordinate) versus linear PI fluorescence (abscissa). The negative control was incubated with a negative mouse Ig and labeled with FITC, and the test case was incubated with a monoclonal cytokeratin antibody (clone MNF116). In all cases the DNA content was labeled with PI. There was only cross-over from PI in the FITC detector, which can be seen in the slope of the dotted compensation trace lines, and no cross-over from FITC in the PI detector, because no slope, in vertical direction, can be seen in the FITC positive counts in the 2C, 4C, 6C, 8C, and 10C clusters in (b). Ideally, the CC would form a straight line, slightly deformed by the logarithmic scaling of the ordinate. In practice the ideal line is scattered due to the influence of auto fluorescence, photon count statistics and noise. Additional peaks of positive counts are visible in the 2C, 4C, 6C, 8C, and 10C clusters in the TEST, which are not included in the CC.

The different compensation values within one sample are illustrated in Figures 3(a) and 3(b) by the two white compensation trace lines. These two illustrations show the uncompensated dot plots of an isotype NC (upper left graph) and TEST (upper right graph) of one of the additional 45 cases. Each graph plots all counts (grey dots) represented by their FITC value (ordinate) as a function of the PI value (abscissa). The common counts are represented by the black dots. The compensation trace line is defined as the best fitting line through the CCs. The dotted compensation trace line is the result of fitting a line through the CC of the NC and SSC (the SSC is not visualized). The full compensation trace line is fitted through the CC of the NC and the TEST values. The visual effect of applying two different compensation values to the test sample from Figure 3(b), is illustrated in Figures 3(c) and 3(d). In Figure 3(c) the compensation result of the TEST is illustrated, after applying the dotted compensation trace line.  Figure 3(d) illustrates the compensated events after applying the straight compensation trace line. The median values of the 2C–10C peaks are illustrated with an “X.” The main visual difference between Figures 3(c) and 3(d) is the distribution of the median values. These medians are distributed along the full compensation trace line.

## 4. Discussion

We have evaluated an automated data-driven approach to fluorescence compensation in a two-color setting. All other compensation methods used thus far involve operator interaction and limit a fully automated data analysis. In order to provide confirmation and validation for the proposed methodology, we have used two methods that are commonly used in flow cytometry laboratories as reference, the Summit and the Winlist software. We have compared the outcome of the proposed DDC method against the outcome of these reference methods. The results show that (1) two color flow cytometry compensation and analysis of sentinel lymph nodes in Summit and DDC lead to comparable results, despite that Summit uses different compensation values than DDC does, (2) the effects of different approaches to calculate compensation values based on the CC can be visualized within one sample, (3) DDC is a data-driven method that combines the information of a NC or SSC with a TEST to calculate sample-specific trace lines, which enables the possibility to automatically analyze large datasets of individual cases batchwise. This is where DDC improves current compensation methods.

According to the Summit reference guide, the Summit software uses a variant of our formulas (1a) and (1b) for the calculation of the compensation values [19]. That variant is stated as

(7a) $$\begin{array}{c} D_{A}=A+S_{BA}B_{\text{true}}, \end{array}$$

(7b) $$\begin{array}{c} D_{B}=B+S_{AB}A_{\text{true}}. \end{array}$$

In these modified formulas (7a) and (7b) there is a difference between the acquired values for *A* and *B* and the unknown true values *A*_true_ and *B*_true_. The equations proposed here make use of the unknown values “*A*_true_” and “*B*_true_.” In the work presented here, we have selected the dye combination such that the initial assumption could be stated as *S*_AB_ = 0. From this limitation it follows that *D_B_* = *B* = *B*_true_ as can be seen from Figure 3(b). Under this specific condition, (7a) can be rewritten to confirm (3a). Therefore, the compensation mathematics in the Summit software should behave exactly like the equations used by DDC. However, our results clearly show that the same compensation value applied to (3a) or to the Summit equation (7a) always resulted in different distributions of compensated counts. This can be observed as a visual difference in the RIDB. This difference in distribution is not explained by the information obtained from the Summit reference guide [19].

To better understand the different distributions in the compensated dot plots, we have recompensated 10 data files using the Winlist software and have compared these results with data obtained using Summit. The Winlist option was chosen as a valuable alternative, as it is an independent, commercially available and commonly used software. The compensation algorithm in Winlist is based also, as we have done in the DDC approach, on (1a) and (1b) [20]. In order to allow a correlation between the sets of compensation values obtained using the Summit and the Winlist software, a rescaling of the Summit compensation units was required to units that are used by Winlist. Appendix B contains the details of this conversion, including the regression analysis. This rescaling of the Summit compensation values by an independent compensation software enabled us to perform a validated comparison with the DDC method using identical compensation units. The observed correlation between the respective compensation values provides clear evidence that the DDC method can replace manual compensation.

The DDC method as proposed in this work makes it possible to objectively compare the effect on fluorescence compensation results when using different approaches. 

In the experiments performed in our laboratory, both FITC and PI are used for simultaneous staining. The compensation performed requires an estimate of the cross-over of signal from the PI dye into the FITC detector (FL1). This cross-over effect and its estimate were also discussed in earlier work [2]. In this work we therefore used a NC and TEST sample setup for the direct comparison between values obtained through the conventional analysis and through the automated calculation via the DDC algorithm.

One alternative path in the literature claims that the percentage cross-over can only be estimated with using an SSC [1, 4, 5]. Incorporation of an SSC, only containing PI, into the DDC algorithm approach was made to also comply with this alternative path. The results as shown in Section 3.3 indicate that using the SSC setup, with DDC, leads to slightly lower compensation values than using the NC setup. This difference in outcome between both approaches is illustrated in Figures 3(c) and 3(d). These two dot plots illustrate the difference in appearance of the compensated counts. The increasing median values along the trace line, marked with an “X,” in Figure 3(c) may suggest under-compensation. However, our results indicate that the RIDB shift between the two compensated plots is caused by the included FITC component in the isotype NC. This FITC component elevates all the counts in the isotype NC, compared to the SSC. This elevation causes the increased compensation value. Therefore on strictly theoretical grounds applying DDC to an isotype NC and TEST leads to overcompensation. However, in this specific situation this will result in a more horizontal distribution of the median values. This horizontal distribution of the median values makes application of a horizontal threshold more suitable for discrimination between FITC positive and negative counts.

Although we found a better distribution of median values after overcompensation, in a specific two color setting, this does not imply that overcompensation always yields better results. When compensated values of one fluorochrome influence the compensated values of other fluorochromes, overcompensation is not recommended. On the other hand when the fluorochromes FITC, PI, and APC are combined, there is only cross-over from PI in FITC and from PI in APC. In this specific setting the compensated FITC and APC values do not influence each other, therefore both can be automatically compensated with DDC, based on two pairs of isotype controls and their TEST.

Batchwise compensation with currently available software is only possible with stable compensation values, which can be monitored by daily calibration and performance tracking. However, PI binds noncovalently to DNA and therefore the intensity of the PI signal is dependant on the cell concentration. Given the same amount of PI, single-cell suspensions with high cell concentrations result in lower PI intensities per cell. The result is a shift of the acquired FL3-a histogram to the left, as compared to samples that contain lower cell concentrations. DNA acquisitions are therefore standardized by adapting the voltage of the PMT of the first acquisition in each tumour sample, until the median value of the first peak falls in channel 200 of a 1024-resolution, linear acquired, DNA histogram [21]. Variable PMT settings between cases lead to case-specific compensation values. The work presented here takes the element of case-specific compensation values into account. The observed correlation of the compensation values between the manual analysis in Summit and the automated DDC analysis clearly shows that the proposed DDC approach can even calculate different compensation values batchwise. This opens the path to complete automated data analysis for DNA phenotype acquisitions. 

The evolution from limited multiparameter to full polychromatic flow cytometry comes with increasing flow cytometry colors and parameters [22]. This leads to increased data output in more dimensions than the human brain can comprehend, and we will therefore have to rely on computers for help with complex matters including compensation. The problem of current compensation algorithms is that they are solely based on mathematics [1]. Alternatively classification algorithms like artificial neural networks (ANNs) support vector machines (SVMs) or *k*-means clustering have the potential to analyze high-dimensional data sets. However they lead to black-box analysis. It is only recently that classification algorithms have been used with integrated operator experience in flow cytometry, either for the selection of a training set supporting ANN-based gating [23] or as a paradigm for automated density based merging of data clusters [24]. 

The DDC approach presented here also integrates operator experience to appropriately deal with sample-specific variables. The set of CC represents the specific area in the traditional approach to set a compensation trace line [2, 10, 25] and can easily be visualized in a dot plot. Visualization of data analysis is of psychological importance to operators and scientists, giving them the possibility to interact, when specific or unexpected distributions appear upon data acquisition. All current methods however require operator intervention, sometimes iterative. This is time consuming, depends on operator experience, and can lead to error, even when expertise is available. Objectivity and quantification are essential parameters in the onset to automation of the process. Based on the work presented, we conclude that DDC improves operator-associated objectivity in fluorescence compensation and facilitates visual representation of data subsets (CC). 

We expect that the implementation of DDC will improve the use of classification algorithms by generating training sets with data that have been corrected for case specific variability.

## Figures and Tables

**Figure 4:**
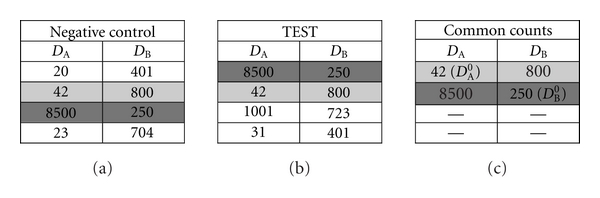
Illustration of the identification of the common counts and the calculation of the cross-over value. Each 2-color experiment consists of 2 acquisitions; a negative control (NC) and a TEST. Matrices are shown for the NC (a) and TEST (b) based on an experiment with a total of 4 counts. Cross-over exists from fluorochrome* B* into detector *D_A_* and from fluorochrome *A* into detector *D_B_*. The matching rows of the NC and TEST matrices (grey) are defined as the matrix of the Common Counts (c). The CCs matrix contains counts with zero primary fluorescence for fluorochromes *A* (*D^0^_A_*) and *B* (*D^0^_B_*). Separation is required into counts with zero primary fluorescence for fluorochrome *A *and counts with zero primary fluorescence for *B*. Cross-over values can be calculated directly from the common count. In this example, *S*_*BA*_ = 42/800 = 0.05 and *S*_*AB*_ = 250/8500 = 0.03.

**Table 1:** The compensation values obtained on 10 data files using Summit and Winlist software. The compensation value corrects for spillover of the PI dye (detected in FL3) into the FL1 detector (FITC).

Compensation value Summit	Compensation value Winlist
1.0	0.05
0.9	0.04
1.35	0.07
1.45	0.08
1.65	0.09
1.50	0.08
1.20	0.07
0.80	0.04
0.80	0.04
1.1	0.05

## Appendices

### A. Formalization of Compensation for 2-Color Flow Cytometry with Double Cross-Over

In the case of 2-color experiments with double cross-over, a NC uses two non relevant antibodies for background staining, one coupled to fluorochrome *A* and one coupled to fluorochrome *B*. In this experiment the CC will contain a combination of counts with zero primary fluorescence for fluorochromes *A* (*D*_*A*_^0^) and *B* (*D*_*B*_^0^) (see Figure 4).

In a dot plot of *D_A_* against *D_B_*, the CC will form two populations with two different slopes *S*_*BA*_ and *S*_*AB*_. With these two populations separated, *S*_*BA*_ can be calculated with the help of (A.1a). Similarly, *S*_*AB*_ can be calculated using the rewritten equation (A.1b):

(A.1a) $$\begin{array}{c} A=\frac{D_{A}^{0}-S_{BA}D_{B}}{1-S_{BA}S_{AB}}=0\Rightarrow D_{A}^{0}-S_{BA}D_{B}=0\Rightarrow S_{BA}=\frac{D_{A}^{0}}{D_{B}}, \end{array}$$

(A.1b) $$\begin{array}{c} B=\frac{D_{B}^{0}-S_{AB}D_{A}}{1-S_{BA}S_{AB}}=0\Rightarrow D_{B}^{0}-S_{AB}D_{A}=0\Rightarrow S_{AB}=\frac{D_{B}^{0}}{D_{A}}. \end{array}$$

### B. Rescaling of the Compensation Values Found in Summit

To compare the manual compensation values generated in the Summit software with those obtained with the DDC algorithm, the compensation units need to have a quantifiable relationship. Unfortunately, the unit of compensation in the Summit software is not documented. To properly define this unit, we have compared the “Summit” units to those units as defined in the Winlist software. This software uses compensation algorithms described by Bagwell. These algorithms have been extensively documented [1–3, 5, 10, 25].

We used 10 of the acquired data files and reanalyzed these using the Winlist software.  Table 1 lists the compensation values obtained with the two software models. The regression analysis of the manual compensation values obtained using the Summit software and the compensation values obtained using Winlist revealed the following linear relation:

(B.1) $$S_{{\text{SUMMIT}}^{*}}=0.062*S_{\text{SUMMIT}}-0.011,$$

where *R*-square = 0.96, RMSE = 0.0040, with *S*_SUMMIT_ being the compensation value as obtained by Summit and *S*_SUMMIT*_ the rescaled compensation value.

## References

[1] Bagwell CB, Adams EG (1993). Fluorescence spectral overlap compensation for any number of flow cytometry parameters. *Annals of the New York Academy of Sciences*.

[2] Stewart CC, Stewart SJ (1999). Four color compensation. *Communications in Clinical Cytometry*.

[3] Roederer M (2002). Compensation in flow cytometry. *Current Protocols in Cytometry*.

[4] Herzenberg LA, Tung J, Moore WA, Herzenberg LA, Parks DR (2006). Interpreting flow cytometry data: a guide for the perplexed. *Nature Immunology*.

[5] Roederer M (2001). Spectral compensation for flow cytometry: visualization artifacts, limitations, and caveats. *Cytometry*.

[6] Leers MP, Schoffelen RH, Hoop JG (2002). Multiparameter flow cytometry as a tool for the detection of micrometastatic tumour cells in the sentinel lymph node procedure of patients with breast cancer. *Journal of Clinical Pathology*.

[7] Leers MP, Schutte B, Theunissen PH, Ramaekers FC, Nap M (1999). Heat pretreatment increases resolution in DNA flow cytometry of paraffin-embedded tumor tissue. *Cytometry*.

[8] Corver WE, Cornelisse CJ, Fleuren GJ (1994). Simultaneous measurement of two cellular antigens and DNA using fluorescein-isothiocyanate, R-phycoerythrin, and propidium iodide on a standard FACScan. *Cytometry*.

[9] Wersto RP, Chrest FJ, Leary JF, Morris C, Stetler-Stevenson M, Gabrielson E (2001). Doublet discrimination in DNA cell-cycle analysis. *Communications in Clinical Cytometry*.

[10] Corver WE, Fleuren GJ, Cornelisse CJ (2002). Software compensation improves the analysis of heterogeneous tumor samples stained for multiparameter DNA flow cytometry. *Journal of Immunological Methods*.

[11] Roederer M, Murphy RF (1986). Cell-by-cell autofluorescence correction for low signal-to-noise systems: application to epidermal growth factor endocytosis by 3T3 fibroblasts. *Cytometry*.

[12] Tung JW, Parks DR, Moore WA, Herzenberg LA, Herzenberg LA (2004). New approaches to fluorescence compensation and visualization of FACS data. *Clinical Immunology*.

[13] Parks DR, Roederer M, Moore WA (2006). A new “logicle” display method avoids deceptive effects of logarithmic scaling for low signals and compensated data. *Cytometry A*.

[14] Balkay L http://www.mathworks.com/matlabcentral/fileexchange/9608-fcs-data-reader.

[15] More JJ, Sorensen DC (1983). Computing a trust region step. *SIAM Journal on Scientific Computing*.

[16] Byrd RH, Schnabel RB, Shultz GA (1988). Approximate solution of the trust region problem by minimization over two-dimensional subspaces. *Mathematical Programming*.

[17] Branch MA, Coleman TF, Li Y (1999). Subspace, interior, and conjugate gradient method for large-scale bound-constrained minimization problems. *SIAM Journal on Scientific Computing*.

[18] Maecker HT, Trotter J (2006). Flow cytometry controls, instrument setup, and the determination of positivity. *Cytometry A*.

[19] DAKO Summit 4.0 reference guide.

[21] Ormerod MG, Tribukait  B, Giaretti W (1998). Consensus report of the task force on standardisation of DNA flow cytometry in clinical pathology. DNA flow cytometry task force of the European society for analytical cellular pathology. *Analytical Cellular Pathology*.

[22] Chattopadhyay PK, Hogerkorp CM, Roederer M (2008). A chromatic explosion: the development and future of multiparameter flow cytometry. *Immunology*.

[23] Quinn J, Fisher PW, Capocasale RJ (2007). A statistical pattern recognition approach for determining cellular viability and lineage phenotype in cultured cells and murine bone marrow. *Cytometry A*.

[24] Walther G, Zimmerman N, Moore W (2009). Automatic clustering of flow cytometry data with density-based merging. *Advances in Bioinformatics*.

[25] Stewart CC, Stewart SJ (2003). A software method for color compensation. *Current Protocols in Cytometry*.

